# Impact of metabolic enzyme activity variability on dabrafenib disposition

**DOI:** 10.3389/fphar.2025.1643618

**Published:** 2025-09-11

**Authors:** Shi-yu Wang, Qing Chen, Zhong-xi Chen, Jing Chen, Jing Yuan, Li-shang Dai, Lian-guo Chen, Xiao-dan Zhang

**Affiliations:** ^1^ Wenzhou Medical University Wenzhou Seventh People's Hospital, Wenzhou, Zhejiang, China; ^2^ School of Pharmaceutical Sciences, Wenzhou Medical University, Wenzhou, Zhejiang, China; ^3^ Department of Pharmacy, The First Affiliated Hospital of Wenzhou Medical University, Wenzhou, Zhejiang, China

**Keywords:** CYP3A4, dabrafenib, loratadine, drug-drug interaction, genetic polymorphism

## Abstract

**Introduction:**

The systemic exposure of dabrafenib correlates with its adverse drug reactions. A thorough understanding of its pharmacokinetic profile is crucial for precise clinical application.

**Methods:**

An optimized liver microsomal incubation system was established to screen for inhibitors of dabrafenib metabolism. Recombinant human CYP3A4 microsomes were prepared using a baculovirus-insect cell expression system. Analytes were quantified using ultra-performance liquid chromatography–tandem mass spectrometry (UPLC–MS/MS). The in vivo relevance of the inhibitory effects was further validated in Sprague-Dawley rats.

**Results:**

Loratadine was identified as the most potent inhibitor, with IC_50_ values of 14.01 ± 2.82 μM in rat liver microsomes and 52.40 ± 4.63 μM in human liver microsomes. It suppressed over 90% of dabrafenib metabolism through mixed-type inhibition. In vivo, co-administration of loratadine significantly increased the systemic exposure of dabrafenib compared to administration of dabrafenib alone. Specifically, the half-life (T_1/2_) and peak concentration (C_max_) increased by 548.65% and 237.43%, respectively, while CLZ/F and VZ/F were markedly reduced. These effects were attributed to inhibition mediated by loratadine. Additionally, CYP3A4 genetic polymorphisms considerably influenced the pharmacokinetics of dabrafenib: the CYP3A4.28 variant exhibited higher intrinsic clearance than the wild-type CYP3A4.1, whereas CYP3A4.8 showed reduced clearance.

**Discussion:**

Both loratadine-mediated drug-drug interactions and CYP3A4 genetic polymorphisms critically alter the metabolism of dabrafenib. Dosage adjustments are necessary when these factors are present concurrently.

## 1 Introduction

Dabrafenib, a selective BRAF V600 mutation kinase inhibitor, is clinically approved as monotherapy or in combination with trametinib (a MEK inhibitor) for multiple indications. These include unresectable/metastatic BRAF-mutated melanoma, metastatic non-small cell lung cancer, BRAF V600E-mutated anaplastic thyroid carcinoma, and pediatric recurrent low-grade gliomas with V600 mutations (Dummer et al., 2020; Busaidy et al., 2022; Chou et al., 2022; Atkins et al., 2023; Bouffet et al., 2023). Substantial interindividual variability in systemic exposure to dabrafenib and its metabolite hydroxy-dabrafenib has been documented in clinical cohorts (Puszkiel et al., 2019; Balakirouchenane et al., 2020). Notably, these pharmacokinetic variations show no significant correlation with demographic factors—including age, gender, or body mass index (BMI)—implicating metabolic enzyme activity and drug-drug interactions as primary determinants (Isberner et al., 2022). Cutaneous toxicity constitutes the most frequently reported adverse effect, manifesting as rash, photosensitivity reactions, and hyperkeratosis (Mackin et al., 2019; Bumbacea et al., 2022). Raman spectroscopy analyses have established a direct correlation between epidermal dabrafenib concentration and skin toxicity severity, supporting local drug accumulation as a mechanistic contributor to cutaneous adverse events (Azan et al., 2017). These findings underscore the necessity of individualized dose optimization to balance therapeutic efficacy with toxicity mitigation.

Interindividual variability in cytochrome P450 (CYP450) enzymatic activity significantly influences drug exposure levels (Harpaz et al., 2010; Thiengsusuk et al., 2020; Yu et al., 2023). Kinetic analyses using substrate depletion assays demonstrate that dabrafenib undergoes extensive metabolism primarily mediated by CYP2C8 and CYP3A4, with minor CYP2C9 involvement (Lawrence et al., 2014). Its major metabolites exhibit distinct clearance pathways: hydroxy-dabrafenib is exclusively metabolized by CYP3A4, while desmethyl-dabrafenib is predominantly cleared by CYP3A4, with additional contributions from CYP2C19 and CYP2C9. Carboxy-dabrafenib, however, remains unaffected by the evaluated CYP enzymes (Lawrence et al., 2014; Puszkiel et al., 2019). Critically, both hydroxy- and desmethyl-dabrafenib undergo further CYP3A4-mediated metabolism, suggesting CYP3A4 modulators (inducers/inhibitors) may substantially alter systemic dabrafenib exposure (Lawrence et al., 2014; Balakirouchenane et al., 2020; Nebot et al., 2021; Sorf et al., 2022). This is supported by pharmacokinetic data: ketoconazole (a potent CYP3A4 inhibitor) coadministration increased dabrafenib C_max_ by 33% and area under curve (AUC) by 71% (Puszkiel et al., 2019; Balakirouchenane et al., 2021).

Despite these insights, the mechanistic basis of dabrafenib’s metabolic regulation remains incompletely characterized, with limited comprehensive pharmacokinetic data available. Dabrafenib exhibits high oral bioavailability (F = 95%) after single-dose administration (Denton et al., 2013). However, repeated dosing induces time-dependent CYP3A4 auto-induction, increasing apparent clearance and reducing plasma exposure (Puszkiel et al., 2019; Balakirouchenane et al., 2021). Although dabrafenib and hydroxy-dabrafenib have been profiled in population pharmacokinetic and drug-drug interactions (DDI) studies (Ellens et al., 2017; Balakirouchenane et al., 2023), further research is needed to fully elucidate the metabolic landscape and clinically relevant interindividual variability in its disposition.

CYP3A4, the predominant CYP450 enzyme responsible for metabolizing 30%–50% of clinical drugs, exhibits distinctive tissue distribution patterns, with highest expression in the liver (constituting 30% of total hepatic CYP content) and intestine (representing 80% of intestinal CYP proteins) (Iversen et al., 2022; Wang et al., 2023). This enzyme demonstrates substantial genetic polymorphism and functional variability (Zanger and Schwab, 2013; Hakkola et al., 2020; Zhou and Lauschke, 2022). However, variant-specific effects on dabrafenib metabolism remain uncharacterized. To address this knowledge gap, we systematically characterized 48 pharmacological agents for drug-drug interaction potential and quantitatively assessed enzymatic kinetics using wild-type CYP3A4.1 alongside 21 genetic variants. This approach provides essential quantitative insights to optimize dabrafenib dosing regimens and advance personalized therapy.

## 2 Materials and methods

### 2.1 Chemicals and reagents

Dabrafenib (purity >98%) and the internal standard (IS) carbamazepine (purity >99%) were purchased from Shanghai Macklin Biochemical Technology Co., Ltd. (China). Hydroxy-dabrafenib was obtained from Toronto Research Chemicals (Canada), and loratadine was acquired from Tianjin Xinsbio Bio-Tech Co., Ltd. (China). Reduced nicotinamide adenine dinucleotide phosphate (NADPH) was supplied by Roche Pharmaceuticals Ltd. (Switzerland). Rat liver microsomes (RLM) and human liver microsomes (HLM) were purchased from Corning Life Sciences Ltd. (United States). HPLC-grade acetonitrile, formic acid, and methanol were purchased from Merck (Germany). All other chemicals and solvents were of analytical grade.

### 2.2 Preparation of RLM

RLM was obtained according to established methods (Wang et al., 2015). Briefly, rat livers were weighed and homogenized in ice-cold phosphate buffer containing 0.01 mmol/L sucrose. The homogenate was centrifuged at 17,000 × g for 15 min at 4 °C. The supernatant was transferred to new tubes and recentrifuged at 17,000 × g for 15 min. The resulting supernatant was then ultracentrifuged at 100,000 × g for 60 min at 4 °C. The microsomal pellet was resuspended in ice-cold 0.01 mmol/L PBS (pH = 7.2–7.4). Protein concentration was determined using a Bradford assay kit (Thermo Scientific, Waltham, MA, United States).

### 2.3 Enzymatic reaction *in vitro*

The 200 µL incubation system contained 0.1 M Tris-HCl (pH = 7.6), 0.5 mg/mL RLM or HLM, 1 mM NADPH, and dabrafenib. After pre-incubation (37 °C, 5 min), reactions were initiated by adding 1 mM NADPH and terminated after 40 min with 300 µL ice-cold acetonitrile. Carbamazepine (100 ng/mL) was added as the IS. Samples were vortex-mixed (2 min), centrifuged (17,000 × g, 10 min, 4 °C), and supernatants analyzed by UPLC-MS/MS. Hydroxy-dabrafenib concentrations were quantified via UPLC-MS/MS. Reaction velocity (*V*) was calculated as hydroxy-dabrafenib formation rate (pmoL/min/µg protein).

For kinetic studies, Michaelis-Menten curves were obtained using dabrafenib concentrations (0.5–200 µM) in RLM and HLM. Samples were processed as above. Curves were plotted (dabrafenib concentration vs. *V*) and fitted nonlinearly (Prism 9, GraphPad).

For drug interaction screening, inhibitors (100 µM final concentration) were screened in the standard incubation system. Compounds showing ≥70% inhibition underwent half-inhibitory concentration (IC_50_) determination. Loratadine IC_50_ was assessed at concentrations (0–100 µM) using substrate concentration of 15 µM in RLM and 4 µM in HLM, respectively. IC_50_ was determined using the following formula: Y = 100/(1 + 10^(X-LogIC_50_)).

For inhibition mechanism studies, kinetic analyses of loratadine inhibition were conducted in both rat and human liver microsomes. In RLM, dabrafenib concentrations spanned 3.75–30 µM with co-administered loratadine (0–28 µM), while HLM assays utilized dabrafenib (1–8 µM) and loratadine (0–50 µM). Michaelis-Menten curves were generated following the methodology detailed above to characterize the interaction kinetics and determine inhibition parameters.

### 2.4 Preparation of recombinant human CYP3A4 and cytochrome B5 baculosomes

Recombinant CYP3A4 and CYPb5 proteins were prepared according to our previously established protocol (Fang et al., 2017). Briefly, dual expression vectors (pFastBac-Dual) were used to construct CYP3A4-CYPOR and CYPb5-CYPOR expression plasmids. Subsequently, the corresponding baculosomes were harvested by ultracentrifugation using the baculovirus-insect cell expression system, and the target proteins were confirmed. Protein expression levels for each type were qualitatively and quantitatively assessed by Western blotting and BCA assay, respectively.

### 2.5 *In vitro* enzyme kinetics assay using CYP3A4 baculosomes

The 200 µL incubation system comprised 0.1 M Tris-HCl, 5 µg CYP3A4.1 or other CYP3A4 variants, 5 µg CYPb5, 1 mM NADPH, and dabrafenib. A dabrafenib concentration of 10 µM was used to evaluate variant specific metabolic activity. Michaelis-Menten kinetics were assessed using dabrafenib concentrations of 5, 10, 20, 50, 100, and 250 µM. Subsequent processing steps followed the procedures outlined in Section 2.3.

### 2.6 Animal experiments

Male Sprague-Dawley rats (220 ± 30 g) obtained from Zhejiang Vital River Laboratory Animal Technology Co., Ltd. were housed under controlled conditions (25 °C ± 2 °C, 60% ± 5% humidity, 12-h light/dark cycle) for a 7-day acclimatization period. All experimental procedures were approved by the Institutional Animal Care and Use Committee of Wenzhou Medical Laboratory Animal Ethics Committee (approval No.: wydw2023-0461). Rats were fasted overnight with free access to water prior to experimentation. Twelve rats were randomly divided into two groups (*n* = 6 per group): Group A received a single oral dose of 31.25 mg/kg dabrafenib, while Group B was administered 5 mg/kg loratadine followed 30 min later by 31.25 mg/kg dabrafenib. Both compounds were suspended in 1% CMC-Na. Animals were given free access to food 4 h post-dosing. Serial blood samples were collected from the tail vein at 0.08, 0.25, 0.5, 1, 2, 3, 4, 6, 12, 24, and 48 h post-dose. Plasma aliquots (50 µL) were combined with 150 µL acetonitrile and 20 µL internal standard (100 ng/mL). After vortex-mixing for 2 min, samples were centrifuged at 17,000 × g for 10 min, and the supernatant was subjected to UPLC-MS/MS analysis.

### 2.7 UPLC-MS/MS conditions

The concentrations of dabrafenib and hydroxy-dabrafenib were quantified by UPLC-MS/MS. Chromatographic separation was performed on a Waters Acquity UPLC BEH C18 column (2.1 mm × 50 mm, 1.7 µm particle size) maintained at 40 °C. The mobile phase consisted of 0.1% aqueous formic acid (A) and acetonitrile (B), delivered at 0.3 mL/min with the following gradient program: 90% A (0–0.15 min), linear transition to 10% A (0.15–0.8 min), 10% A (0.8–1.5 min), and return to 90% A (1.5–2 min). Total run time was 2 min. Detection employed a Waters Xevo TQS triple quadrupole mass spectrometer (Milford, MA, United States) operated in positive-ion multiple reaction monitoring mode. Quantification transitions were m/z 520.10 → 307.20 for dabrafenib, m/z 536.20 → 323.10 for hydroxy-dabrafenib, and m/z 237.10 → 194.10 for IS.

### 2.8 Molecular docking

The 2D structures of dabrafenib and loratadine were retrieved from the PubChem database (https://pubchem.ncbi.nlm.nih.gov), while the CYP3A4 crystal structure was obtained from the RCSB Protein Data Bank (PDB). Structural optimization was performed using PyMOL 3.0.4. Hydrogen addition and charge assignment were conducted with AutoDockTools 1.5.7. Molecular docking simulations were executed in PyRx to calculate binding energies. Protein-ligand complexes were aligned and visualized using PyMOL.

### 2.9 Statistical methods

Michaelis-Menten kinetics, IC_50_ values, and mean plasma concentration-time curves were calculated and plotted using GraphPad Prism 9.0. Noncompartmental analysis was performed with pharmacokinetic software (DAS, version 3.0; Bontz Inc., Beijing, China) to determine pharmacokinetic parameters. One-way ANOVA compared wild-type CYP3A4.1 parameters against other variants, while unpaired t-tests assessed differences between experimental groups. Data are presented as mean ± standard deviation, with statistical significance defined as *P* < 0.05.

## 3 Results

### 3.1 Dabrafenib-based CYP3A4 inhibitor screening in RLM systems reveals potent inhibition by loratadine

**FIGURE 1:**
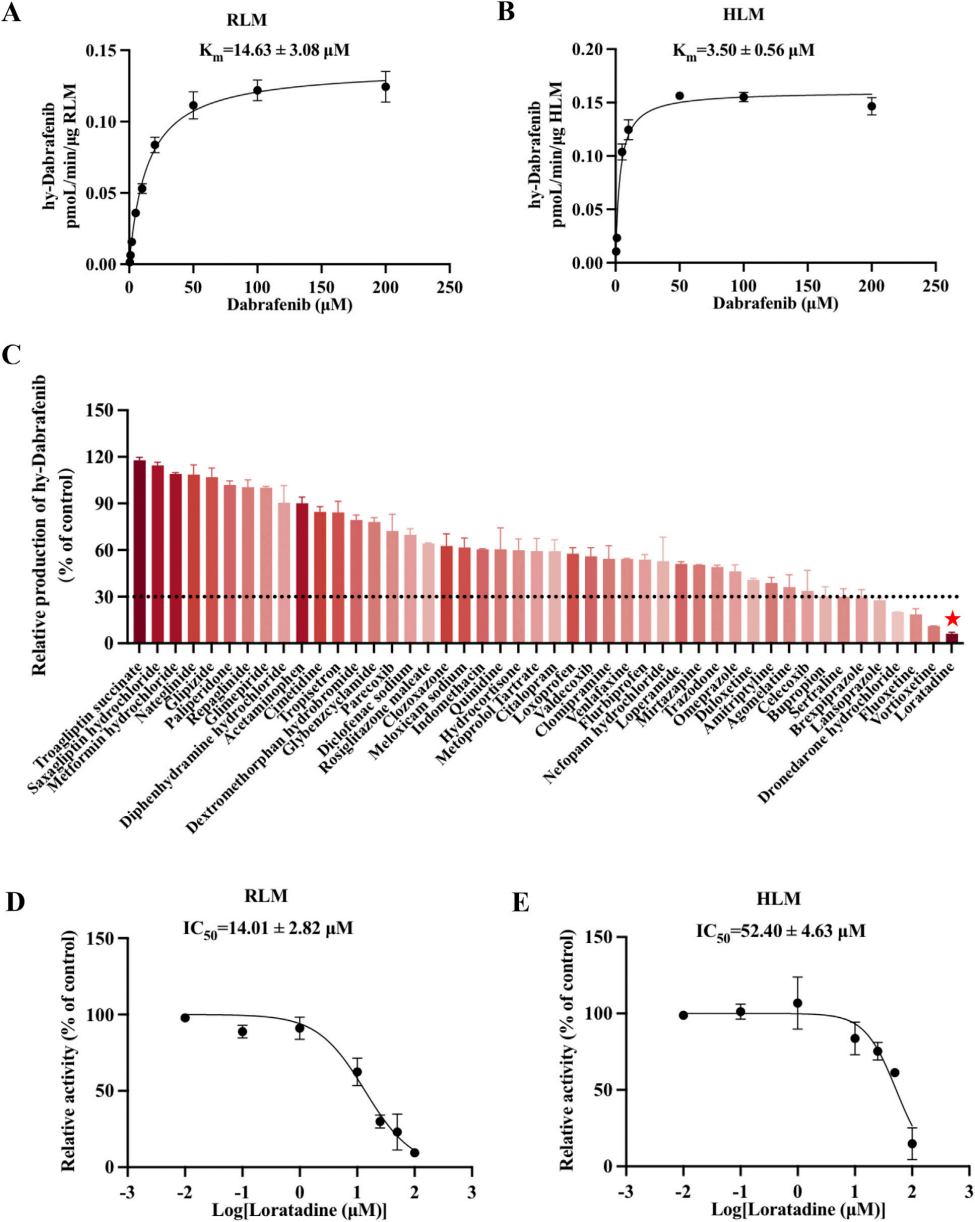
Loratadine inhibits the metabolism of Dabrafenib. **(A,B)** Incubation was conducted using RLM and HLM. Michaelis-Menten curves were generated through nonlinear fitting in GraphPad Prism 9, with dabrafenib concentration as the x-axis and velocity (V, normalized to RLM protein content) as the y-axis. **(C)** Screening of candidate compounds in RLM. Hydroxy-dabrafenib concentrations were quantified by LC-MS/MS. The percentage of control activity was calculated and plotted. **(D,E)** IC_50_ values of loratadine were determined at concentrations ranging from 0.01 to 100 μM in both RLM and HLM enzyme systems. Data were presented as mean ± SD, *n* = 3.

Utilizing an established enzyme incubation system, hydroxy-dabrafenib concentrations were quantified and Michaelis-Menten curves constructed for dabrafenib metabolism in RLM and HLM. As shown in Figures 1A,B, kinetic analyses revealed dabrafenib parameters of K_m_ = 14.63 ± 3.08 µM and V_max_ = 0.14 ± 0.01 pmoL/L/min/µg in RLM, versus K_m_ = 3.50 ± 0.56 µM and V_max_ = 0.16 ± 0.001 pmoL/L/min/µg in HLM. Subsequent screening of 48 drugs at RLM K_m_ concentrations (Figure 1C; Supplementary Table S1) identified eight compounds-including sertraline, brexpiprazole, lansoprazole, dronedarone hydrochloride, fluoxetine, vortioxetine, and loratadine-exhibiting >70% inhibition of dabrafenib metabolism. Loratadine demonstrated the strongest inhibition (>90%), representing a previously unreported interaction with dabrafenib. To confirm this finding, loratadine’s inhibitory potency was assessed in both systems, yielding IC50 values of 14.01 ± 2.82 μM (RLM) and 52.40 ± 4.63 μM (HLM) (Figures 1D,E).

### 3.2 Loratadine altered the major pharmacokinetic characteristics of dabrafenib in rats

**FIGURE 2:**
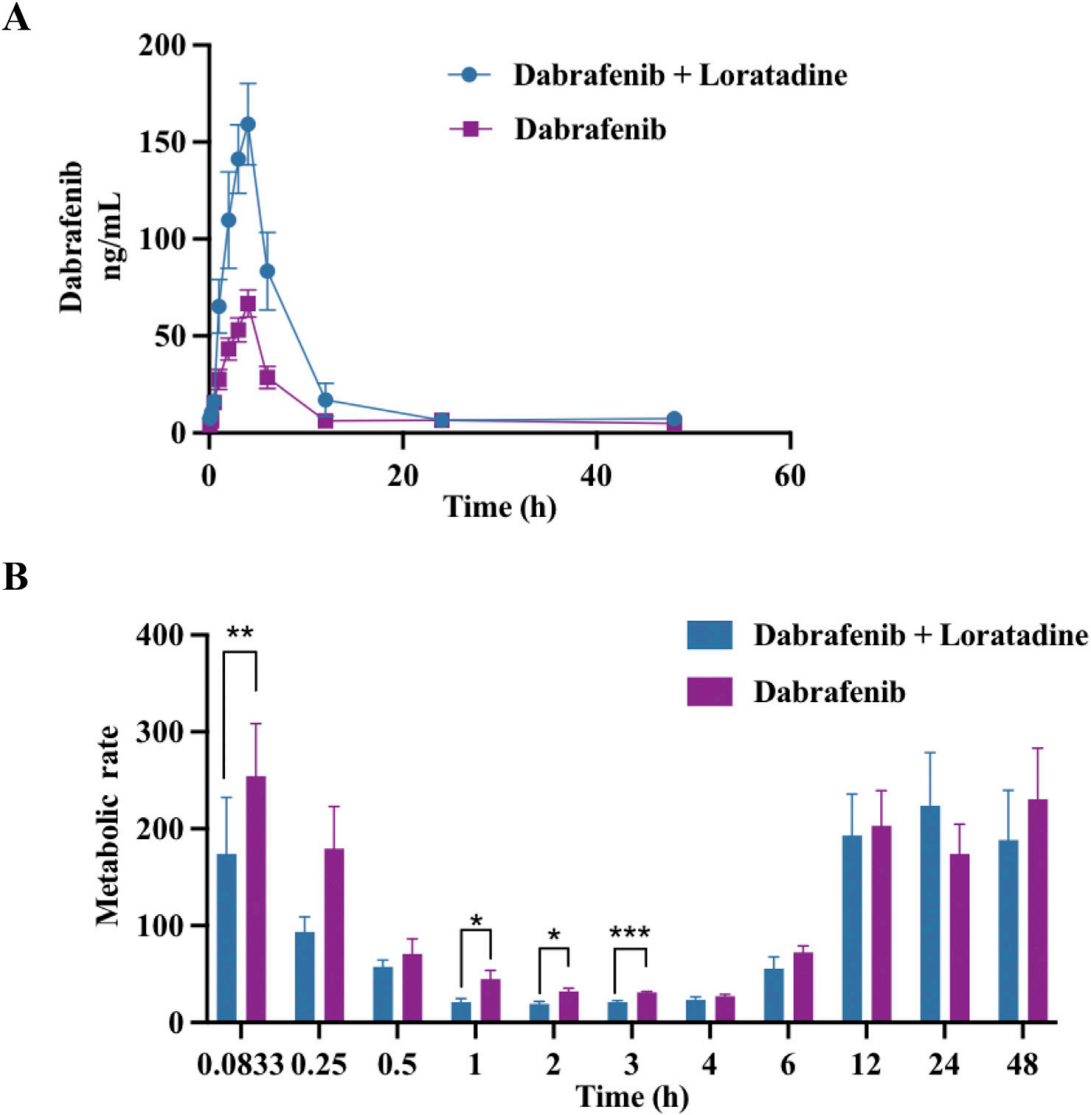
Effect of loratadine coadministration on the pharmacokinetics of dabrafenib in rats. **(A)** Mean plasma concentration–time profiles of dabrafenib following administration alone or in combination with loratadine. **(B)** Relative effects of loratadine coadministration on dabrafenib metabolism across different blood collection time points. Data were presented as mean ± SD, *n* = 6. *P < 0.05, ***P < 0.001.

Given loratadine’s potent *in vitro* inhibition, we evaluated its effects on dabrafenib pharmacokinetics *in vivo*. To eliminate potential confounding from estrous cycle hormonal fluctuations, only male rats were used. Animals were fasted pre-experiment to control for dietary influences. As shown in Figure 2A and Table 1, coadministration of loratadine significantly altered dabrafenib pharmacokinetics: AUC_(0-t)_ and AUC(0- ∞ ) increased by 212.27% and 157.55%, respectively. Additionally, t_1/2_ and C_max_ rose by 548.65% and 237.43%, respectively, while CL_z/F_ and V_Z/F_ decreased substantially. Additionally, the plasma concentration-time profile of the metabolite hydroxy-dabrafenib is presented in Supplementary Figure S1. The metabolic ratio was calculated based on the respective concentrations of hydroxydabrafenib and dabrafenib. Results demonstrated that the metabolic ratio (hydroxydabrafenib to dabrafenib) around T_max_ was significantly lower in the combination group than in the dabrafenib-alone group (Figure 2B).

**TABLE 1:** Key pharmacokinetic parameters of dabrafenib following administration to Sprague-Dawley rats.

Parameters	Dabrafenib + loratadine	Dabrafenib
AUC_(0-t)_ (ng/mL*h)	1211.47 ± 344.86	570.71 ± 170.96**
AUC(0- ∞ ) (ng/mL*h)	1213.81 ± 345.19	770.42 ± 293.11*
t_1/2_ (h)	5.55 ± 0.81	30.45 ± 14.99**
T_max_ (h)	4.33 ± 1.51	3.83 ± 0.41
V_z/F_ (L/kg)	224.55 ± 78.59	1843.67 ± 723.94**
CL_z/F_ (L/h/kg)	27.87 ± 9.28	45.84 ± 17.04*
C_max_ (ng/mL)	159.93 ± 39.34	67.36 ± 14.20**

Note: AUC, area under the concentration-time curve; t_1/2_, elimination half-life; T_max_, time to peak concentration; V_z/F_, apparent volume of distribution; CL_z/F_, blood clearance rate; C_max_, maximum blood concentration. *P < 0.05, **P < 0.01.

### 3.3 Mechanistic insights into loratadine-mediated inhibition of dabrafenib metabolism

**FIGURE 3:**
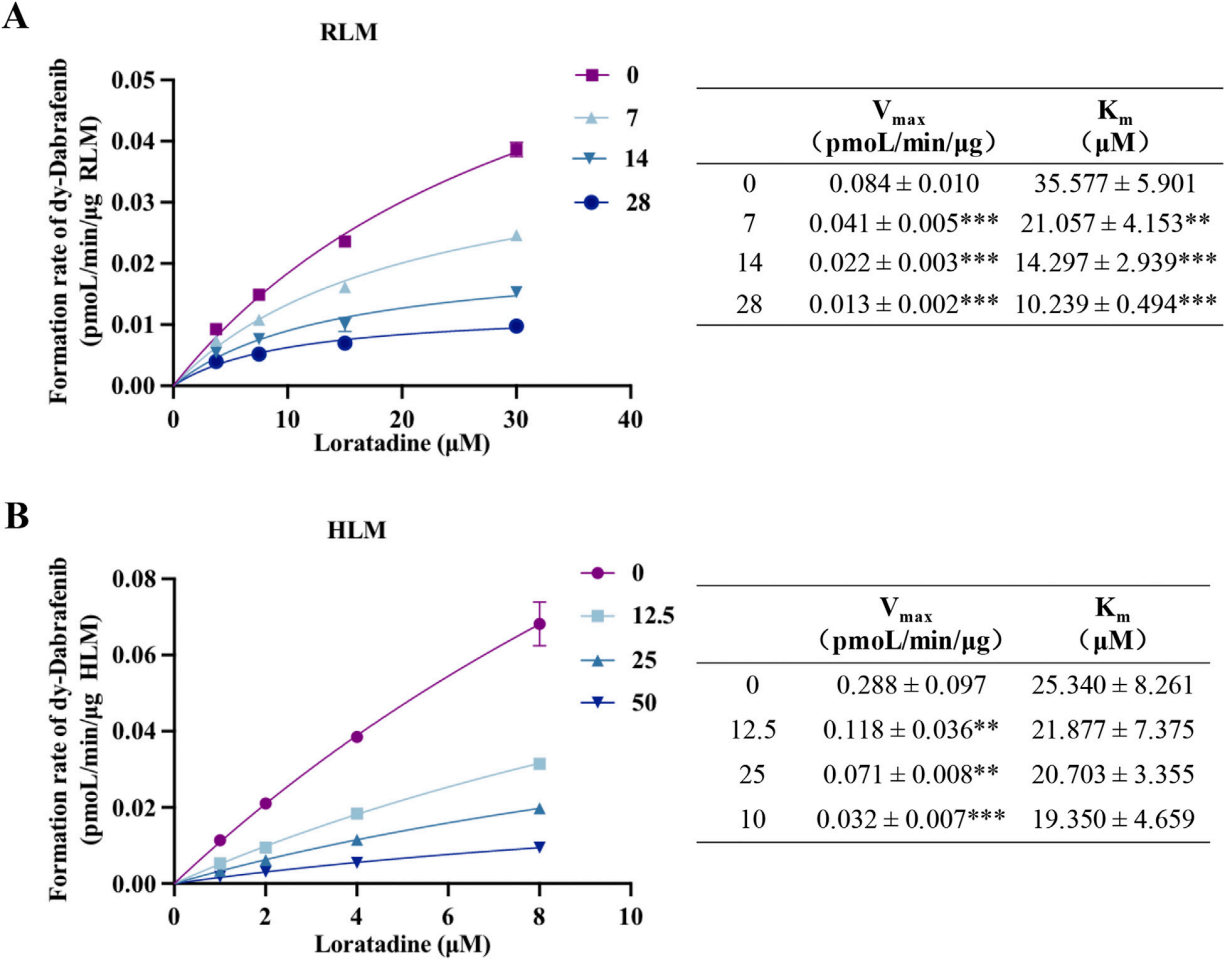
Mechanism of loratadine-mediated inhibition of dabrafenib metabolism. **(A,B)** Nonlinear regression analysis showing the effect of loratadine on the Michaelis–Menten kinetics of dabrafenib in RLM and HLM. The type of inhibition was determined by evaluating changes in K_m_ and V_max_. Data were presented as mean ± SD, *n* = 3. **P < 0.01, ***P < 0.001.

Kinetic analyses and molecular docking studies elucidated the inhibitory mechanism of loratadine on dabrafenib metabolism and its steric hindrance of CYP3A4 binding. Nonlinear fitting of RLM and HLM data demonstrated concentration-dependent decreases in V_max_ and K_m_ values for dabrafenib (Figures 3A,B), with proportional declines characteristic of mixed-type inhibition. Complementary enzyme kinetic modeling (Supplementary Figure S2) confirmed loratadine inhibits dabrafenib metabolism through a mixed mechanism involving both non-competitive and uncompetitive components.

**FIGURE 4:**
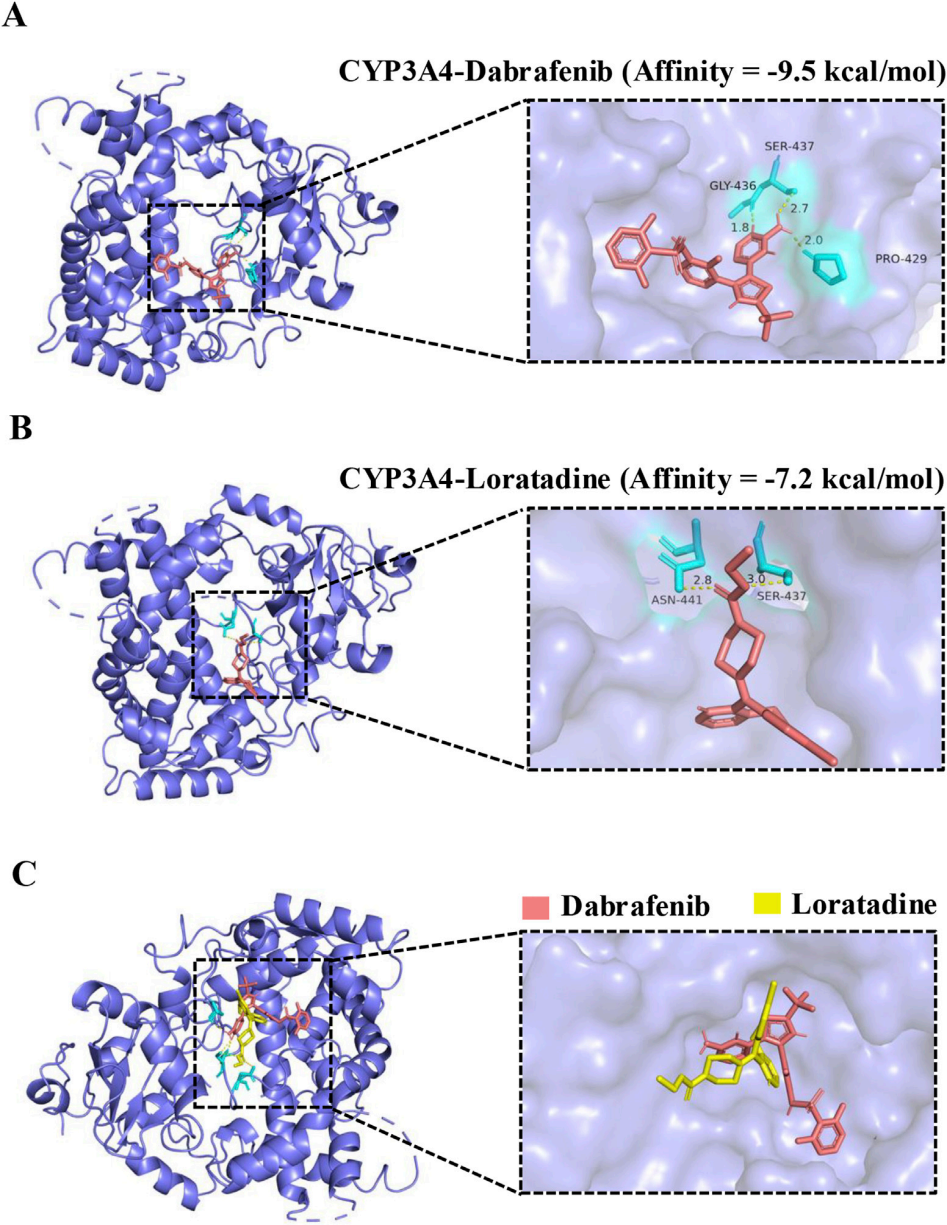
Molecular docking of dabrafenib and loratadine with CYP3A4. **(A)** Predicted binding mode of dabrafenib within the CYP3A4 active site. **(B)** Predicted binding mode of loratadine within the CYP3A4 active site. **(C)** Structural alignment of loratadine superimposed onto the dabrafenib-CYP3A4 binding complex.

Molecular docking revealed dabrafenib forms hydrophobic interactions with CYP3A4 residues SER437, GLY436, and PRO429 (binding energy: 9.5 kcal/mol), forming an active pocket (Figures 4A,B). Loratadine similarly engaged ASN441 and SER437 via hydrophobic interactions (−7.2 kcal/mol). Crucially, loratadine occupied a distinct binding site on CYP3A4 (Figure 4C), inducing conformational changes that sterically hinder dabrafenib binding and disrupt its productive interaction with the enzyme.

### 3.4 Impact of CYP3A4 genetic polymorphisms on dabrafenib metabolism

**FIGURE 5:**
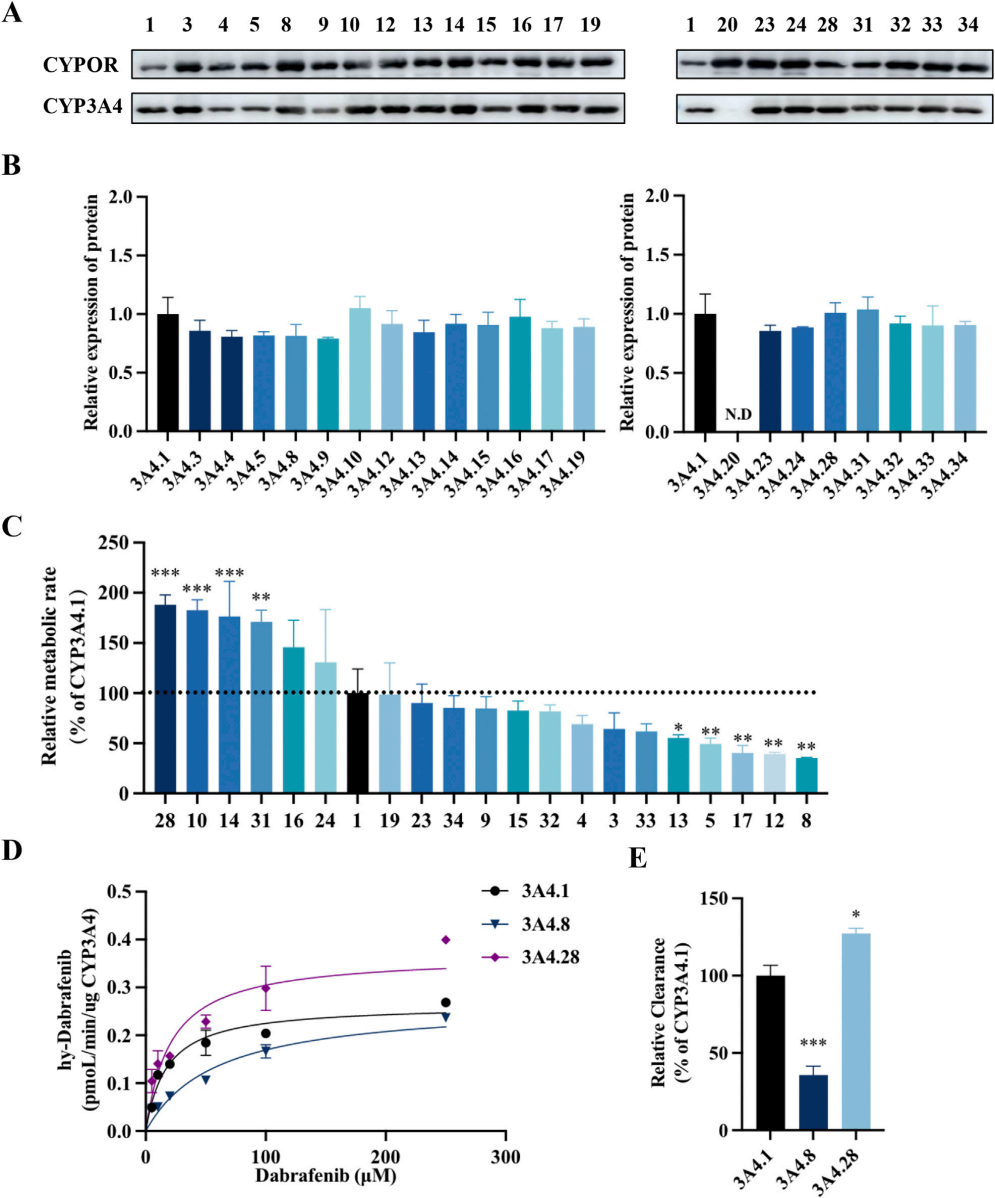
Impact of CYP3A4 genetic polymorphisms on the metabolic profile of dabrafenib. **(A)** Western blot analysis and corresponding densitometric quantification of CYP3A4 protein expression. **(B)** Densitometric quantification of immunoblots shown in panel A. **(C)** Enzyme kinetic assays of recombinant CYP3A4 variants. Metabolic activity of each variant toward dabrafenib is expressed relative to that of the wild-type CYP3A4.1. **(D)** Michaelis–Menten curves were generated by nonlinear regression (GraphPad Prism 9) with dabrafenib concentration (5–250 μM) as the independent variable and metabolic velocity (V, normalized to CYP content) as the dependent variable. **(E)** Apparent V_max_ and K_m_ values derived from Michaelis–Menten analysis. Relative metabolic clearance of each variant is shown compared to CYP3A4.1. Data represent mean ± SD, *n* = 3. *P < 0.05, **P < 0.01, ***P < 0.001.

CYP3A4, the primary enzyme responsible for the oxidative metabolism of dabrafenib, exhibits genetic polymorphisms that can influence its metabolic efficiency. To assess the impact of these genetic variations, the effects of various CYP3A4 variants on dabrafenib enzyme kinetics were investigated. Specifically, 22 human CYP3A4 baculosomes representing distinct ethnic backgrounds were selected and prepared for this study, as depicted in Figures 5A,B. As shown in Figure 5C, CYP3A4 genetic polymorphisms significantly affected the relative extent of dabrafenib metabolism. Based on relative metabolic activity compared to wild-type CYP3A4.1, the variants were categorized into four groups (Table 2): (1) Enhanced activity: CYP3A4.28, 10, 14, and 31, exhibiting significantly increased metabolic activity (171.27%–189.47% of CYP3A4.1); (2) Wild-type-like activity: CYP3A4.16, 24, 19, 23, 34, 9, 15, 32, 4, 3, 33, and 13; (3) Reduced activity: CYP3A4.13, 5, 12, and 8.; (4) Minimal activity: CYP3A4.20, exhibiting nearly complete loss of activity.

**TABLE 2:** The impact of CYP3A4.1 and other CYP3A4 variants on the production of hydroxy-dabrafenib.

Variants	Production of hydroxy-dabrafenib (µM)
3A4.1	0.0038 ± 0.0009
3A4.3	0.0024 ± 0.0006
3A4.4	0.0026 ± 0.0003
3A4.5	0.0019 ± 0.0002*
3A4.8	0.0013 ± 0.0001**
3A4.9	0.0032 ± 0.0004
3A4.10	0.0071 ± 0.0007***
3A4.12	0.0015 ± 0.0001**
3A4.13	0.0021 ± 0.0001
3A4.14	0.0067 ± 0.0013***
3A4.15	0.0031 ± 0.0004
3A4.16	0.0055 ± 0.0010
3A4.17	0.0015 ± 0.0003**
3A4.19	0.0037 ± 0.0012
3A4.20	ND
3A4.23	0.0034 ± 0.0007
3A4.24	0.0050 ± 0.0020
3A4.28	0.0072 ± 0.0008***
3A4.31	0.0065 ± 0.0004**
3A4.32	0.0031 ± 0.0003
3A4.33	0.0023 ± 0.0003
3A4.34	0.0032 ± 0.0004

Note: Compared to CYP3A4.1, *P < 0.05, **P < 0.01, ***P < 0.001. ND, not detected.

Representative variants CYP3A4.8 and CYP3A4.28 were selected for further enzyme kinetic analysis of dabrafenib. Michaelis-Menten kinetics analysis (Figures 5D,E) revealed that compared to CYP3A4.1, CYP3A4.8 showed a significantly increased K_m_, while CYP3A4.28 exhibited a significantly increased V_max_. Consequently, the relative clearance of CYP3A4.8 decreased by 66.67%, whereas that of CYP3A4.28 increased by 26.67% (Table 3). Collectively, these data underscore the significant influence of CYP3A4 genetic polymorphisms on dabrafenib metabolism.

**TABLE 3:** CYP3A4.1, CYP3A4.8, and CYP3A4.28 mutations on the metabolic kinetic parameters of Dabrafenib.

Variants	V_max_ (pmoL/L/min/μg CYP3A4)	K_m_ (μM)	CL_int_ (V_max_/K_m_) (μL/min/μg CYP3A4)
3A4.1	0.266 ± 0.007	17.78 ± 1.535	0.015 ± 0.001
3A4.8	0.247 ± 0.030	47.37 ± 12.486**	0.005 ± 0.001***
3A4.28	0.362 ± 0.026**	19.59 ± 1.624	0.019 ± 0.001**

Note: Compared to CYP3A4.1, **P < 0.01, ***P < 0.001.

## 4 Discussion

In this study, we conducted an *in vitro* screening of 48 drugs—including antihistamines, antipyretic analgesics, hypoglycemic agents, gastrointestinal drugs, and antidepressants—to evaluate their effects on dabrafenib metabolism. All screened agents carry clinical relevance for potential co-administration with dabrafenib: antidepressants (e.g., sertraline, mirtazapine) for mood disorders; proton pump inhibitors (PPIs, e.g., omeprazole, lansoprazole) for acid reflux; NSAIDs/COX-2 inhibitors (e.g., celecoxib, diclofenac) for pain management; and antidiabetic agents (e.g., glipizide, metformin) for hyperglycemia control. High-risk scenarios include concomitant use with strong CYP2C8 inhibitors (e.g., clopidogrel-proxy quinidine) or CYP3A4 inhibitors (e.g., fluoxetine), which may elevate dabrafenib exposure and toxicity. The inclusion of metabolic probes such as dextromethorphan (CYP2D6) and chlorzoxazone (CYP2E1) further assessed enzymatic specificity, while widely used agents (e.g., acetaminophen, loratadine) reflect real-world polypharmacy patterns. Thus, this screening panel integrates mechanistic breadth with clinical utility for predicting DDIs during dabrafenib therapy. Among these, eight compounds—involving sertraline, brexpiprazole, lansoprazole, dronedarone hydrochloride, fluoxetine, vortioxetine, and loratadine—exhibited >70% inhibition of dabrafenib metabolism. The identification of these potent inhibitors provides critical data for clinical precautions regarding dabrafenib combination therapies.

Allergic reactions represent a commonly reported adverse event associated with dabrafenib (Choi et al., 2020). To mitigate these effects, loratadine, a second-generation H_1_ receptor antagonist and non-sedating antihistamine with established clinical efficacy in managing dermatological allergies (Hunto et al., 2020; AlMasoud et al., 2022), was proposed for co-administration. Its favorable safety profile and demonstrated therapeutic efficacy position it as a viable option for alleviating dabrafenib-induced hypersensitivity reactions while minimizing sedative side effects. Furthermore, loratadine had been proposed as a CYP3A4 inhibitor, suggesting a potential pharmacokinetic interaction with dabrafenib (Li et al., 2010). Investigation revealed loratadine exhibited the highest inhibition extent (>90%), a finding with significant clinical implications. Consequently, given the potential for co-administration, exploration of the interaction between loratadine and dabrafenib warranted investigation.

*In vitro* experiments confirmed that loratadine significantly inhibited dabrafenib metabolism through a mixed-type inhibition mechanism, involving both non-competitive and uncompetitive components. This inhibitory pattern was consistent in both RLM and HLM. However, we observed notably divergent IC_50_ values for loratadine between them. The underlying cause of this discrepancy was not explicitly investigated in the present study. Based on prior research findings, the observed IC_50_ differences are likely attributable to interspecies variations in relative enzyme activities (Zhang et al., 2020). From a clinical relevance perspective, the IC_50_ values obtained in HLM exceed the blood exposure levels of single-dose dabrafenib in humans, suggesting potential inhibitory effects may occur clinically (Puszkiel et al., 2019). However, the magnitude of inhibition could be modulated by tissue-specific drug distribution (e.g., intestinal/skin exposure), metabolizer phenotypes (e.g., CYP3A4 poor/rapid metabolizers), and chronic dosing cumulative effects. To further elucidate the molecular basis of this interaction, we employed molecular docking to simulate the binding of CYP3A4 with dabrafenib and loratadine. The results studied further demonstrated that dabrafenib bonded to CYP3A4 residues through hydrophobic interactions. In contrast, loratadine occupied a distinct binding site, inducing conformational changes that sterically hindered dabrafenib-CYP3A4 interactions and altered the substrate selectivity and structural specificity of dabrafenib metabolism.

*In vivo* experiments demonstrated that loratadine significantly altered the pharmacokinetic profile of dabrafenib in rats. Co-administration of dabrafenib with loratadine resulted in substantial increases in AUC_(0-t)_, AUC(0- ∞ ), C_max_, and t_1/2_, alongside significant decreases in CL_z/F_ and V_Z/F_. The observed reduction in V_z/F_ likely stemmed from enhanced bioavailability (via gastrointestinal CYP3A4 inhibition) and diminished hepatic first-pass metabolism, consistent with prior evidence that metabolic drug interactions minimally affected distribution volume (Sodhi et al., 2021; Murata et al., 2023). These alterations elevated plasma dabrafenib concentrations, increasing the risk of toxicity due to accumulation. These findings implied that co-administration with loratadine led to elevated plasma exposure, increased bioavailability, reduced apparent clearance, slower metabolism, and potential drug accumulation, which may heighten the risk of adverse reactions due to elevated plasma concentrations. Clinical pharmacokinetic studies demonstrated that a single 150 mg oral dose of dabrafenib achieved C_max_ of 2,160 ng/mL Although the measured IC_50_ values (52.40 ± 4.63 µM in HLM) substantially exceeded therapeutically relevant free plasma concentrations (≈6.3 nM), a comprehensive clinical risk assessment required consideration of: tissue-specific exposure profiles, metabolic enzyme phenotypes, accumulation potential during chronic dosing (Puszkiel et al., 2019; Balakirouchenane et al., 2021).

CYP3A4 plays an integral role in drug metabolism and exhibits considerable genetic polymorphism, resulting in diverse pharmacogenetic phenotypes that influence individual drug response (Guttman et al., 2019; Šemeláková et al., 2021; Zhang et al., 2024). Unlike other cytochrome P450 enzymes, CYP3A4 currently lacks published pharmacogenetic guidelines, primarily due to its complex enzymatic kinetics. This complexity stems partly from the enzyme’s large active site pocket, which accommodates a wide range of substrates with variable metabolic kinetics (Guengerich et al., 2021; Feng et al., 2024). This structural heterogeneity complicates the use of substrate probes and underscores the need for continued research into novel substrates to better elucidate CYP3A4’s pharmacogenetic landscape (Ducharme et al., 2021).

CYP3A4 serves as the primary enzyme responsible for the oxidative metabolism of dabrafenib. Given the significant genetic polymorphism of CYP3A4, variations in dabrafenib blood exposure levels among individuals could lead to divergent metabolic outcomes. However, the impact of CYP3A4 genetic polymorphisms on dabrafenib metabolism remained poorly characterized. This investigation utilized wild-type CYP3A4.1 as a benchmark to assess the functional impacts of 21 CYP3A4 variants on dabrafenib metabolism. Detailed enzyme kinetic analyses were performed on representative variants CYP3A4.8 and CYP3A4.28. Compared to wild-type CYP3A4.1, CYP3A4.8 exhibited significantly diminished metabolism, reflected in a substantially reduced relative clearance, while CYP3A4.28 demonstrated increased clearance. These findings suggest that patients harboring slow-metabolizing variants may require consideration of dose reduction or alternative medications during dabrafenib therapy to mitigate the risk of serious adverse reactions. Conversely, patients with fast-metabolizing variants may risk sub-therapeutic exposure. Additionally, CYP3A4.20, a known non-functional allele, was confirmed to lack catalytic activity towards dabrafenib, consistent with previous reports (Riera et al., 2018; Liu et al., 2023). Collectively, these data highlight the profound impact of CYP3A4 genetic polymorphisms on dabrafenib metabolism and underscore the necessity of genotype-guided dosing strategies to optimize therapeutic efficacy and minimize adverse effects.

## 5 Conclusion

This study systematically investigated the drug interaction profile of dabrafenib, revealed that loratadine exerted significant inhibitory effects on its metabolism in both *in vitro* and *in vivo* settings. Furthermore, CYP3A4 genetic polymorphisms significantly altered dabrafenib metabolic clearance, which highlighted the complex interplay between drug-drug interactions and pharmacogenetic variability in dabrafenib metabolism. Our findings highlight the necessity of genotyping CYP3A4 variants and avoiding loratadine co-administration to optimize dabrafenib dosing and minimize toxicity risks.

## Data Availability

The original contributions presented in the study are included in the article/Supplementary Material; further inquiries can be directed to the corresponding authors.
