# Supplementary material for: Impact of metabolic enzyme activity variability on dabrafenib disposition
Source: Front Pharmacol. 2025 Sep 11;16:1643618. doi: 10.3389/fphar.2025.1643618 (PMC12460146; doi:10.3389/fphar.2025.1643618)
Supplement: Supplementary file 1 [file Supplementaryfile1.docx]

***Supplementary information***

Supplementary Table S1

Supplementary Figure S1

Supplementary Figure S2

**Supplementary Table S1** The information about the 48 drugs.

| **Name** | **CAS** | **Molecular weight** | **Relative activity (% of control)** |
| --- | --- | --- | --- |
| Valdecoxib | 181695-72-7 | 314.36 | 56.01% ± 0.06 |
| Agomelatine | 138112-76-2 | 243.30 | 36.20% ± 0.08 |
| Amitriptyline | 549-18-8 | 313.86 | 38.88% ± 0.04 |
| Bupropion | 31677-93-7 | 276.20 | 30.23% ± 0.06 |
| Venlafaxine | 99300-78-4 | 313.87 | 54.22% ± 0.004 |
| Brexpiprazole | 913611-97-9 | 433.57 | 29.48% ± 0.05 |
| Fluoxetine | 56296-78-7 | 345.79 | 18.63% ±0.04 |
| Trazodone | 25332-39-2 | 408.32 | 49.14% ± 0.01 |
| Mirtazapine | 61337-67-5 | 265.35 | 50.53% ±0.001 |
| Sertraline | 79559-97-0 | 342.69 | 29.92% ± 0.05 |
| Citalopram | 59729-32-7 | 405.3 | 59.29% ± 0.07 |
| Vortioxetine | 508233-74-7 | 279.34 | 11.14% ±0.001 |
| Duloxetine | 136434-34-9 | 333.88 | 40.82% ± 0.01 |
| Clomipramine | 303-49-1 | 314.85 | 54.39% ± 0.08 |
| Dronedarone hydrochloride | 141625-93-6 | 593.224 | 20.07% ± 0.02 |
| Lansoprazole | 103577-45-3 | 369.36 | 27.68% ± 0.01 |
| Paliperidone | 144598-75-4 | 426.48 | 101.95% ± 0.03 |
| Loperamide | 34552-83-5 | 513.50 | 51.15% ± 0.01 |
| Omeprazole | 73590-58-6 | 345.42 | 46.24% ±0.04 |
| Cimetidine | 51481-61-9 | 252.34 | 84.61% ± 0.03 |
| Quinidine | 56-54-2 | 324.42 | 60.50% ± 0.14 |
| Metoprolol Tartrate | 56392-17-7 | 342.41 | 59.44% ± 0.08 |
| Glipizide | 29094-61-9 | 445.54 | 106.96% ± 0.06 |
| Metformin hydrochloride | 1115-70-4 | 165.62 | 109.07% ± 0.01 |
| Glybenzcyclamide | 10238-21-8 | 494.00 | 78.13% ± 0.03 |
| Glimepiride | 93479-97-1 | 490.62 | 100.17% ± 0.01 |
| Saxagliptin hydrochloride | 709031-78-7 | 351.88 | 114.45% ± 0.02 |
| Rosiglitazone maleate | 155141-29-0 | 473.5 | 64.26% ± 0.004 |
| Nateglinide | 105816-04-4 | 317.42 | 108.55% ± 0.06 |
| Repaglinide | 135062-02-1 | 452.59 | 100.54% ± 0.05 |
| Troagliptin succinate | 1029877-94-8 | 475.48 | 117.88% ± 0.02 |
| Diphenhydramine hydrochloride | 147-24-0 | 291.82 | 90.45% ± 0.11 |
| Loratadine | 79794-75-5 | 382.88 | 6.26% ± 0.01 |
| Dextromethorphan hydrobromide | 6700-34-1 | 370.32 | 79.28% ± 0.03 |
| Tropisetron | 89565-68-4 | 284.35 | 84.28% ± 0.07 |
| Acetaminophen | 103-90-2 | 151.16 | 90.08% ± 0.04 |
| Loxoprofen | 68767-14-6 | 246.3 | 57.60% ± 0.04 |
| Parecoxib | 1709956-95-5 | 333.79 | 72.34% ± 0.11 |
| Nefopam hydrochloride | 23327-57-3 | 289.8 | 52.87% ± 0.15 |
| Diclofenac sodium | 15307-79-6 | 318.13 | 69.82% ± 0.04 |
| Celecoxib | 169590-42-5 | 381.37 | 33.72% ± 0.13 |
| Clozoxazone | 95-25-0 | 169.57 | 62.62% ± 0.08 |
| Hydrocortisone | 50-23-7 | 362.47 | 70.48% ± 0.08 |
| Lornoxicam | 70374-39-9 | 371.82 | 65.67% ± 0.04 |
| Dexamethasone | 50-02-2 | 392.47 | 59.84% ± 0.07 |
| Meloxicam sodium | 71125-38-7 | 351.4 | 61.62% ± 0.06 |
| Flurbiprofen | 5104-49-4 | 244.26 | 59.91% ± 0.03 |
| Indomethacin | 53-86-1 | 357.79 | 60.51% ± 0.004 |

**
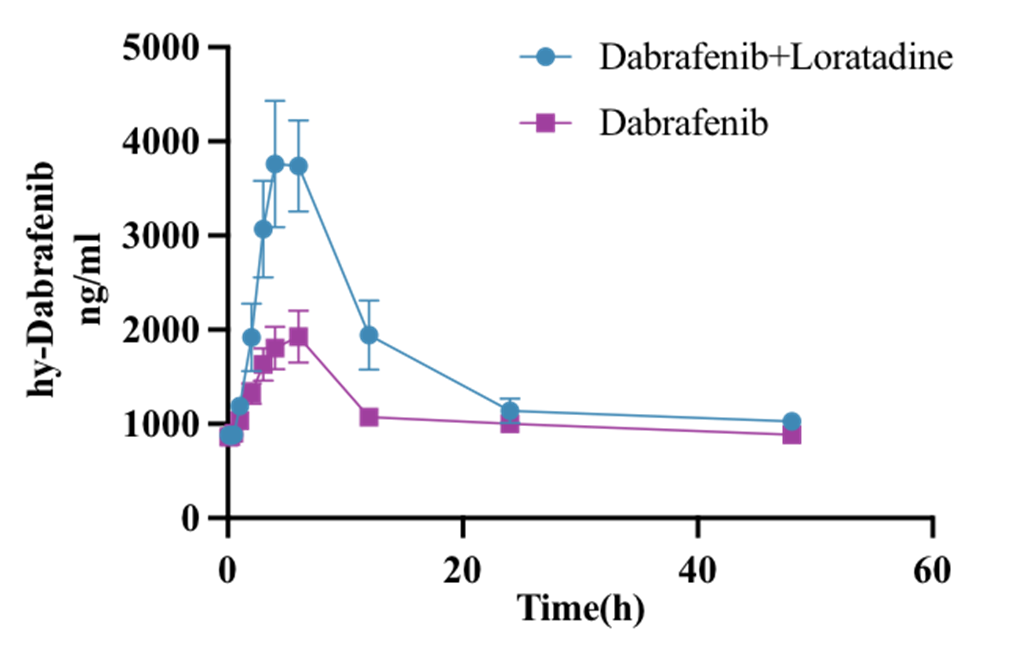
Supplementary Figure S1. The impact of co-administration or non-co-administration of Loratadine on hydroxy-Dabrafenib in rats.** The graph was plotted with blood collection time on the x-axis and blood drug concentration on the y-axis. Curves for Dabrafenib administered alone or in combination with Loratadine are shown.


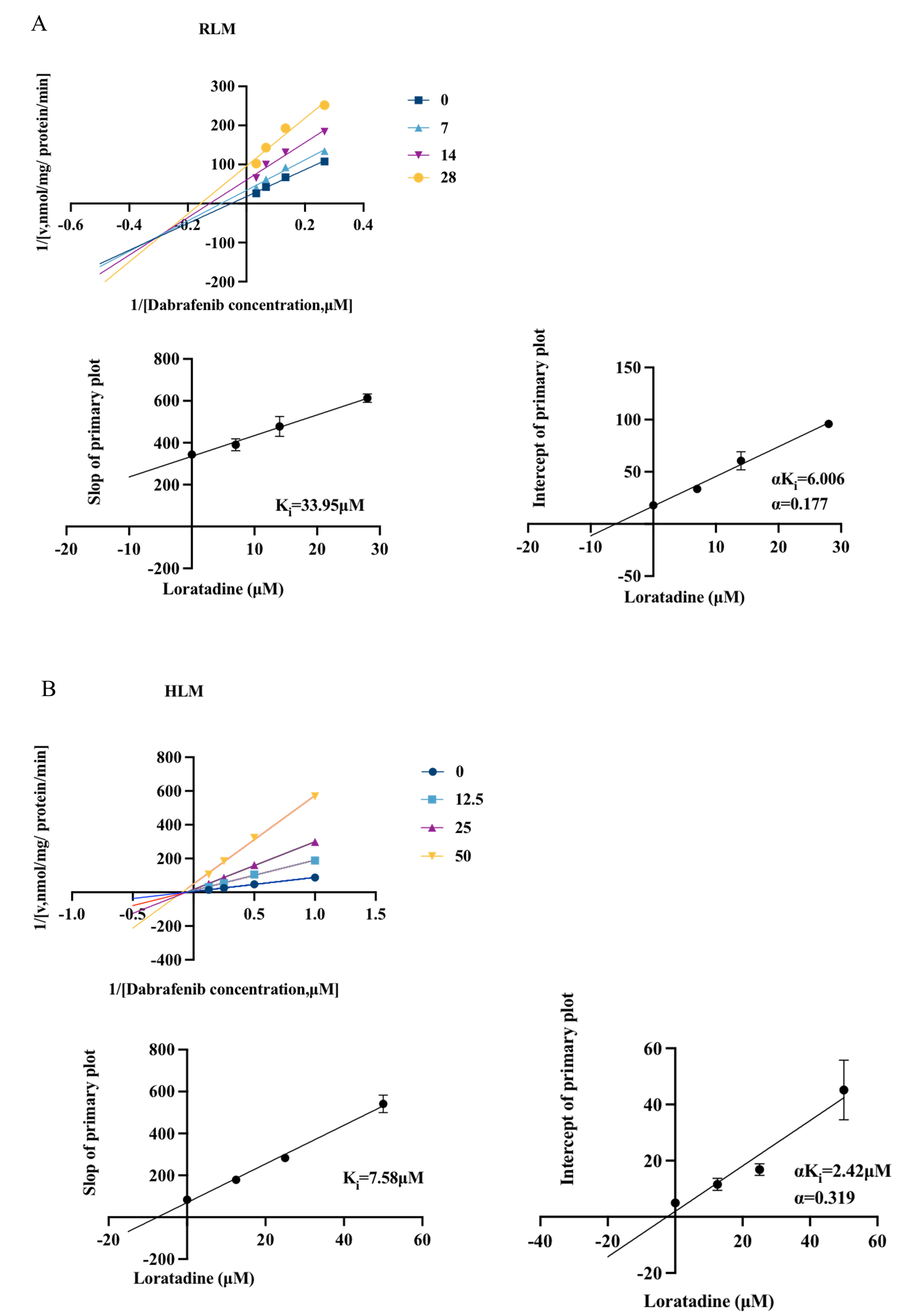


**Supplementary Figure S2. Loratadine inhibited the enzyme kinetics model of Dabrafenib metabolism.** (A-B) The data displayed in the Lineweaver-Burk plot, the secondary plot of Ki, and the secondary plot of αKi show the inhibitory effects of Loratadine at different concentrations on Dabrafenib metabolism in RLM and HLM.
